# Class- and subject teachers’ self-efficacy and emotional stability and students’ perceptions of the teacher–student relationship, classroom management, and classroom disruptions

**DOI:** 10.1186/s40359-021-00606-6

**Published:** 2021-07-08

**Authors:** Alexander Wettstein, Erich Ramseier, Marion Scherzinger

**Affiliations:** Department of Research and Development, University of Teacher Education Bern, Bern, Switzerland

**Keywords:** Classroom discipline, Classroom management, Emotional stability, Interpersonal perception, Self-efficacy, Teacher–student relationship

## Abstract

**Background:**

Teacher self-efficacy and emotional stability are considered crucial resources for coping with classroom demands. We examined how class and subject teachers’ self-efficacy beliefs and emotional stability are related to teachers' and students' perceptions of the teacher–student relationship, classroom management, and classroom disruptions.

**Methods:**

In a sample of eighty-two swiss german 5th and 6th grade classes, 1290 students, their class teacher (*N* = 82), and a selected subject teacher (*N* = 82) filled out a questionnaire assessing classroom disruptions, teacher–student relationships, and classroom management. In a first step, we conducted t-tests on whether class teachers and subject teachers differ in their self-efficacy beliefs and emotional stability. In a second step, we explored by correlation analyses the relations between teacher self-efficacy in classroom management and emotional stability and the teachers’ and students’ perceptions of classroom disruptions, teacher–student relationships, and classroom management. In a third step, we examined by stepwise multiple regression analyses to what extent psychological variables predict teacher perceptions after controlling for students’ ratings, representing rather “objective” classroom features.

**Results:**

In class teachers, high self-rated emotional stability and self-efficacy are associated with a more positive appraisal of teacher–student relationships and classroom management skills (compared with student ratings). By contrast, in subject teachers, high self-efficacy beliefs are associated with a more favorable perception of classroom disruptions, teacher–student relationships, and classroom management, from both the teachers' and students' perspectives.

**Conclusions:**

The results of the present study show a distinctive pattern for class teachers and subject teachers. In class teachers, high self-rated emotional stability and self-efficacy are associated with a more positive evaluation (compared to student ratings) of the teacher–student relationship and classroom management skills but not teacher perceptions of student misbehavior. On the contrary, subject teachers' firm self-efficacy beliefs are associated with more favorable perceptions of classroom characteristics, both from the teachers' and students' perspectives.

**Supplementary Information:**

The online version contains supplementary material available at 10.1186/s40359-021-00606-6.

## Background

The present study examines how teacher self-efficacy and emotional stability are related to teachers' and students' perceptions of classroom disruptions, teacher–student relationships, and classroom management. The majority of research on teacher self-efficacy in classroom management relies solely on teacher ratings of student misbehavior. Only a few studies take into account both teacher and student perspectives (e.g., [1]). Furthermore, as far as we know, there are no studies on self-efficacy that consider the different roles of class teachers and subject teachers.

### Teaching is demanding

Teaching is a very demanding task. Teachers have to cope with highly complex social situations that involve many students, in which events happen simultaneously and often take unpredictable turns [2, 3]. Simultaneously they have the task to organize and structure the classroom to trigger successful teaching–learning processes and foster students' social, emotional, and cognitive development, to promote meaningful learning and student growth [4, 5].

These high demands may cause teacher stress. Within the classroom, teacher stress arises primarily from social-psychological aspects of education, such as difficulties with classroom management and problematic teacher–student relationships, rather than from instructional teaching problems [6]. Studies show that one of the significant strains in the teaching profession are classroom *disruptions* [7, 8]. When faced with a classroom disruption, the teacher has to react immediately, and the whole class witnesses the teacher’s actions [2]. Dealing with classroom disruptions is one of the most salient sources of stress experienced by teachers within the classroom [8]. High classroom demand levels (e.g., classroom disruptions) become particularly stressful when teachers appraise the demands as exceeding their resources for *coping.* Following [9], stress is not an external event itself but rather an interpretation and response to a potential threat. A psychosocial situation is stressful when it was appraised as such [10]. Lazarus and Folkman [9] postulated that such stress appraisal has two stages: primary appraisal and secondary appraisal. In the primary appraisal stage, potential threats, the demands of the situation, and goals and values are evaluated. In the secondary appraisal stage, the resources to deal with those requirements are assessed. Thus, according to transactional models of stress, teachers are vulnerable to stress when they appraise their coping resources as insufficient for classroom demands [11]. Teachers' perceived incompetence in managing student misbehavior leads to higher levels of stress [8].

### Teacher self-efficacy and emotional stability are considered crucial resources

*Teacher self-efficacy and emotional stability are considered crucial resources* for coping with classroom demands [12]. They may buffer the adverse effects of classroom stressors such as classroom disruptions [11] and facilitate coping with these stressful events [13]. Thus, in research on teacher strain, a high sense of teacher self-efficacy and emotional stability are considered central prerequisites for successful teaching and teacher health preventing teacher exhaustion [13].

*Self-efficacy* can be defined as "beliefs in one's capabilities to organize and execute the course of action required to produce given attainments" [14]. In the beginning, teacher self-efficacy was regarded as an overall, fixed construct, as a global personality trait. There is a growing consensus that these beliefs are very context-specific and related to specific activities [15, 16]. Consequently, researchers examined efficacy in critical subareas and developed different scales for the assessment of teacher efficacy. The Scale for Measuring Teacher Efficacy in Classroom Management and Discipline [17] distinguishes three subscales: classroom management and discipline, external influence, and, finally, personal teaching efficacy. The Interpersonal Self-Efficacy Scale [15] comprises the subscale perceived self-efficacy in classroom management. Self-efficacy in classroom management is defined as "teachers' confidence in their capabilities to manage student behavior to achieve order and cooperation in the classroom" [15]. The Ohio State Teacher Efficacy Scale (OSTES) [18] covers the three efficacy for instructional strategies subscales: (e.g., “To what extent can you use a variety of assessment strategies?”), efficacy for classroom management (e.g., “How much can you control disruptive behavior in the classroom?”), and efficacy for student engagement (e.g., “How can you get students to believe they can do well in school work?”).

*Emotional stability* refers to the resistance to psychological distress or the ability to cope with stress. In contrast, emotional instability [19] is a common predictor of teacher exhaustion [20]. Individuals low in emotional stability tend to express more negative emotions and may generally apply nonfunctional coping strategies. Ineffective coping strategies like denial or distancing themselves from the problem make them even more vulnerable to burnout [21]. Teachers with low levels of emotional stability are prone to psychological stress and easily experience unpleasant emotions such as anger, anxiety, depression, and vulnerability. Emotionally unstable individuals tend to interpret neutral or even positive social encounters as threatening and have difficulties handling even minor frustrations [22]. Empirical findings showed that severity ratings of undesirable student behaviors were associated with high conscientiousness and emotional instability. Kokkinos et al. [22] found a positive association between emotional instability and severity ratings for interpersonal sensitivity behaviors. It may be that individuals low in emotional stability feel more interpersonally challenged by children who are more suspicious, distrustful, and sensitive than other children.

Studies show that one of the major strains in the teaching profession are classroom *disruptions* [23]. They can impede the teaching and learning process in many ways and are considered a significant risk factor for teacher exhaustion [7, 8]. Classroom disruptions are defined against the backdrop of an interactional perspective as disruptions of the teaching–learning process [24]. Disruptions in the classroom may emanate from students, as well as from teachers. Nonaggressive (agitation, cutting in) and aggressive student disruptions (threatening, excluding) and lack of organization of the instruction or even aggressive behavior of the teacher (shaming, ridiculing) impair teaching and learning processes. Classroom disruptions can extend over the entire methodological-didactic setting and lead to a working atmosphere marked by many interruptions and restlessness. Classroom disruptions may lead to the emotional exhaustion of the teacher and negatively affect instruction quality, teacher–student relationships, and student achievement [25].

Positive *teacher–student relationships* and good classroom management performance are crucial factors in preventing classroom disruptions [23]. Classrooms are inherently social contexts. Developing and maintaining good *teacher–student relationships* is essential to both preventing classroom disruptions and fostering student learning [4]. Positive teacher–student relationships make a unique contribution to students' social and cognitive development. Appropriate teacher–student relationships are characterized by a rather high degree of teacher influence and proximity to students [26]. Therefore, teachers should establish caring relationships with students and create settings in which students feel secure to explore and learn [27]. Positive teacher–student relationships are related to several positive social, emotional, and learning outcomes [4]. Teachers' socioemotional support is one of the strongest correlates of student adjustment [28, 29] and reduces children’s risk factors [30]. Students misbehave less when relationships with their teachers are positive, and a good teacher–student relationship prevents classroom disruptions [31, 32].

Good *classroom management* performance is one of the most substantial factors preventing classroom disruptions. Classroom management is broadly defined as "the actions teachers take to create an environment that supports and facilitates both academic and social-emotional learning” [33]. Classroom organization is generally perceived as a domain of classroom processes related to how well teachers manage students’ behavior and instructional time and whether they provide lessons and materials that maximize learning opportunities [27].

### Class and subject teachers

In Switzerland, already at the primary level (primary school), students are taught by a class teacher and different subject teachers. At the primary level, in contrast to secondary levels I and II, there is a more definite role-specific division between class teachers and subject teachers. The class teacher bears the primary responsibility for the class, introduces classroom rules, takes on most of the teaching, and is the primary contact person for students, parents, and school authorities. Students are also taught by subject teachers, who teach individual subjects to this class. So far, only a few studies are available on the teaching of subject teachers at the primary level. Observational studies [34] and questionnaire-based studies [35] indicate that more classroom disruptions generally occur in classes given by subject teachers than in those of class teachers. By their different roles in the classroom, students and teachers may perceive classroom processes differently. Any of these different perspectives may have specific benefits and disadvantages.

How do teachers perceive teacher–student relationships, classroom management, and classroom disruptions? It can be assumed that teachers' perceptions are influenced by two different sources. On the one hand, their ratings may mirror objective features of the classroom, like those perceived by students, which represent rather objective classroom features. On the other hand, the teachers' ratings may also be influenced by psychological variables such as their self-efficacy beliefs and self-assessed emotional stability.

Teacher self-efficacy and emotional stability can basically fulfill two functions. At best, these valuable coping resources have a real "objective" impact on teacher behavior, reflected in the students' perception of classroom characteristics. In the worst case, teacher self-rated self-efficacy and emotional stability merely serve as a "subjective" lens through which teachers evaluate their teaching behavior without impacting their behavior and, consequently, without affecting students' perception. However, this is not an all-or-nothing question. It can be expected that teacher perception is influenced gradually by both "objective" and psychological factors, depending on the construct that is measured.

Some studies emphasize rather “*objective” influences* of emotional stability and teacher self-efficacy on teachers’ behavior in the classroom. Hüfner [36] suggest that, potentially, “teachers’ perceived ability to cope with challenging students may partly determine which classroom management behaviors, strategies, and styles they ultimately adopt.” Results from studies of the consequences of teacher self-efficacy in classroom processes indicate that high-efficacy teachers “[…] tend to effectively cope with a range of problem behaviors; use proactive, student-centered classroom behavior strategies; and establish less conflictual relationships with students” [36]. Several studies show that teachers with high self-efficacy report coping more effectively with student misbehavior [37–39]. Hüfner [36] state that they use more proactive behavioral management strategies. Teachers who express high self-efficacy beliefs report being more tolerant of problematic students, less likely to perceive children as problematic, and less likely to exclude students with behavior problems from their class [36]. Also, they tend to be more patient, make better use of class time, criticize students less, encourage student autonomy and responsibility, and persist longer when dealing with challenging students [40, 41].

In contrast, teachers with low classroom management self-efficacy beliefs are more likely to give up easily when faced with disruptive behavior, believing that their actions have little influence [11, 15]. However, these results are based solely on teacher perception, and it remains an open question if teachers’ high self-efficacy beliefs also have positive effects on their behavior in the classroom. This teacher behavior could be indirectly made accessible by assenting students’ perceptions of teacher behavior. Emmer and Hickman [17] found a positive correlation between teacher efficacy in classroom management and self-reported preference for positive strategies (*r* = .30; *p* = .05). However, no significant association was found between teachers' self-perceptions and the judgment of external observers. Based on the apparent lack of correspondence between the judgment of teachers and that of other observers, the authors formulated the following thesis: "It may be that for these teachers, high self-efficacy is a form of denial and permits them to avoid the negative feelings that an honest self-assessment could produce" [17]. Following this point of view, teacher self-efficacy and emotional stability could be conceptualized not as having any objective, measurable influence on teaching behavior but rather as *psychological variables* that represent only self-serving bias and a subjective, benevolent lens through which teachers perceive classroom processes more positively, without any impact on classroom processes. Following this point of view, teacher self-efficacy and emotional stability could represent the lens through which teachers view their classroom environments and students [1]. A high sense of self-efficacy and emotional stability would lead to a more positive perception of classroom features and therefore protect teachers against stress [42]. However, following this line of argumentation, we would not expect any influence of high teacher-efficacy and emotional stability on students’ ratings of classroom characteristics.

*Teachers’ and the students’ perspectives differ in their perception of classroom processes.* Due to their training and professional experience, teachers potentially have the pedagogical-didactic expertise for a valid assessment of the classroom [43]. The complexity and simultaneousness of the processes taking place in the class, however, make self-assessment difficult. Besides, it cannot be ruled out that teachers' judgments are subject to self-serving biases, which put the instruction in a positive light [44]. *The students’ perspective* has a series of advantages. Students observe lessons from a perspective that is mostly free of the burden of action and thus have, unlike the teacher, an observation advantage [45]. Students judge their teachers based on a broad base of experience over many class hours. In most cases, students' perceptions are more consistent with the observations of external observers than are teachers' judgments [46].

### Research questions and hypotheses

1. *Class and subject teachers’ self-efficacy beliefs (Research Question 1).* Do class and subject teachers vary in their self-efficacy beliefs and emotional stability? We expect that class teachers and subject teachers do not differ significantly in their self-efficacy beliefs and emotional stability (Hypothesis 1). Both class teachers and subject teachers evaluate their self-efficacy based on their specific roles in the class. Subject teachers are aware that they have limited contact with the class. They know that they do not have the same influence as class teachers. Therefore, they rate their self-efficacy against the backdrop of their specific role.
2. *Teachers’ judgments (Research Question 2).* How are teacher beliefs about their self-efficacy and emotional stability related to their perception of classroom disruptions, teacher–student relationships, and classroom management? For class teachers, we expect that a high sense of teacher efficacy and emotional stability is associated positively with their judgment of classroom management, teacher–student relationships, and disruptions of the methodological-didactic setting. These domains are predominantly under the control of the teacher. By contrast, we do not expect any relation with aggressive and nonaggressive student behavior, which emanates predominantly from the students (Hypothesis 2a). We presume a similar pattern for the subject teacher, except for their ratings for classroom management. Subject teachers have only a minor influence on the classroom management practices of a given class. Rules are established primarily by class teachers, whereas subject teachers must adapt themselves to the already established classroom management rules of the class teacher. Furthermore, subject teachers are less familiar with individual students. Both factors may hinder efficient classroom management. We consequently do not expect any relation between subject teachers' self-efficacy beliefs and their perceptions of classroom management (Hypothesis 2b).
3. *Students’ judgments (Research Question 3).* How are teacher beliefs about their self-efficacy and emotional stability related to students' perceptions of classroom disruption, teacher–student relationship, and classroom management? It is hypothesized that teachers' self-efficacy and emotional stability do not merely represent self-serving bias and at least partially reflect positive classroom features. We expect that these objective classroom features show up in student ratings to some degree. Consequently, we expect positive correlations between class teachers' self-efficacy, emotional stability, and students' perceptions of teacher–student relationships and classroom management, as well as negative association with classroom disruptions (Hypothesis 3a). We assume a similar pattern for subject teachers. However, due to their limited influence on establishing classroom rules, we do not expect significant correlations for classroom management (Hypothesis 3b).
4. *To what extent do psychological variables predict teacher perceptions? (Research Question 4).* We expect two different sources of influence on teacher perceptions. On the one hand, these ratings may mirror objective features of the classroom, like those perceived by the students, which represent predominantly objective classroom features. On the other hand, psychological variables may also influence teacher ratings, such as their self-efficacy beliefs and self-assessed emotional stability. We expect that both subjective and objective factors of classroom features contribute to teacher perceptions. However, we hypothesize a more considerable influence of psychological variables on constructs, which from the teacher's perspective, depending on their teaching behavior. Regarding the different roles of the class and subject teachers, especially in classroom management, we expect two different patterns of influence of teacher-efficacy and emotional stability on the different perceptions. For class teachers, we predict an association between teacher-efficacy and emotional stability and their perception of setting disruptions, classroom management, and teacher–student relationships. In contrast, we do not expect any influence on their judgment of nonaggressive and aggressive student disruptions (Hypothesis 4a). For subject teachers, we expect an effect on their perception of setting disruptions and teacher–student relationships. We assume not any association for their evaluation of classroom management (which is primarily under the control of the class teacher) and nonaggressive and aggressive student disruptions (Hypothesis 4b).

## Methods

### Participants

In a questionnaire study, 1290 students (48.2% girls, *M*_*age*_ = 11.47 years, *SD* = 0.77), their class teachers (*N* = 82, 64.6% female, *M*_*age*_ = 39.4 years, *SD* = 11.82, *M* = 20.0 lessons taught per week to the class, *SD* = 4.64, *M* = 23.8 lessons taught per week at the school, *SD* = 4.63) and their subject teachers (*N* = 82, 76.8% female, *M*_*age*_ = 42.2 years, *SD* = 10.88, *M* = 6.65 lessons taught per week to the class, *SD* = 3.58, *M* = 16.6 lessons taught per week at the school, *SD* = 7.27) filled out a questionnaire assessing classroom disruptions, teacher–student relationships, and classroom management (Additional file 1).

### Instruments

#### Teacher self-efficacy

This study measures self-efficacy as a task-specific construct reflecting teachers' beliefs in coping with classroom disruptions [47]. The four items focus on the task-specific expectations of teachers toward coping with classroom disruptions: "I can do a lot to prevent disruptions through adaptive classroom organization." "Due to my experience with classroom disruptions, I can manage even difficult classroom situations." "I am sure I can reach even disruptive students when I try." "I know that I can gauge comprehension, even of disruptive students." Internal consistency of this scale ranges from .64 for class teachers to .72 for subject teachers.

#### Emotional stability

Emotional stability was assessed globally by three items: "I often feel tense and nervous." "I quickly resign myself to lack of success." "I can handle disappointments." [19, 48]. The reliability of this three-item scale was .61 for class teachers and .68 for subject teachers.

#### Classroom disruptions, teacher–student relationships, and classroom management

Classroom disruptions were measured using a newly developed questionnaire [49]. This instrument differentiates three distinctive types of disruptions: (1) disruptions of the methodological-didactic setting, (2) nonaggressive student behavior, and (3) aggressive student behavior. Furthermore, the instrument includes (4) *teacher–student relationship* and (5) *classroom management*. All constructs were assessed on a four-point Likert scale from the teacher’s and student’s points of view. The complete teacher and student items are documented in [49]. In the following, the scales are presented using exemplary example items (cf. Table 1).

**Table 1:** Scales with sample items from the student version

Scales	Number of Items	Student version	*α*
SET	4	In the classroom of this teacher, there are a lot of disruptions	.87
NON	4	Some kids talk while this teacher is explaining something	.88
AGS	4	Children kick other children	.88
REL	6	I like this teacher	.95
CLA	3	This teacher has the overview about what's going on in the classroom	.82

SET = methodological-didactic setting disruptions; NON = nonaggressive disruptions by students; AGS = aggressive disruptions by students; REL = teacher–student relationship; CLA = classroom management. In the entire questionnaire development, we strived to formulate items as similar as possible in the teachers’ and students’ versions. For the relationship scale, however, far-reaching changes were necessary. Here, teachers rate the classes, while students rate their teacher (e.g., “I like my class” and “I like this teacher,” respectively)

### Ethical considerations

The study was conducted following the principles of the Helsinki Declaration and the Swiss Federal Act on Data Protection (FADP). The institutional ethics committee of the University of Teacher Education Bern, Commission for Research and Development (KFE) approved the study. We obtained active written consent from all participants as well as their legal representatives. All data was anonymized from the beginning, protecting the identity of all the individual participants.

### Procedure

For the study, we approached 728 German-speaking fifth and sixth-grade classes via the school headmasters. A total of *N* = 86 classes took part in the survey (11.8%). Of these, 30 were fifth-grade classes, 27 sixth-grade classes, and 29 mixed-grade classes in which fifth- and sixth-grade pupils are taught together. In addition to the class teacher, we selected one subject teacher per class. This selection was not representative but based on their willingness to participate. However, we ensured that the selected subject teachers did not hold any class teaching positions. Trained research administered the questionnaires between January and June 2014 during the regular school lessons. 1341 students completed the questionnaire (83.3%). However, 16.7% of the students did not participate in the study. Of these, 13.5% had given their consent but were attending special classes during the survey, were ill, or had changed school. 3.2% of the students (or their parents) did not consent to participate in the study. Of the 86 participating classes, we had to exclude four (*n* = 3 missing data of the subject teachers; *n* = 1 incomplete data on emotional stability and self-efficacy), resulting in a final sample of 82 classes.

### Data analysis

In a first step, we conducted t-tests on whether class teachers and subject teachers differ in their self-efficacy beliefs and emotional stability. In a second step, we explored by correlation analyses the relations between teacher self-efficacy in classroom management and emotional stability and the teachers’ and students’ perceptions of classroom disruptions, teacher–student relationships, and classroom management. In a third step, we examined by stepwise multiple regression analyses to what extent psychological variables predict teacher perceptions after controlling for students’ ratings, which represent rather “objective” classroom features.

## Results

### Class and subject teachers’ self-efficacy beliefs (Research Question 1)

Class teachers have slightly higher scores in their self-efficacy beliefs (Cohen’s *d* = .223, *p* = .123) and emotional stability (*d* = .213, *p* = .167) than subject teachers. However, these small differences do not reach statistical significance. Thus, class teachers and subject teachers do not differ significantly in their perceptions of self-efficacy and emotional stability. This result is in line with Hypothesis 1.

### Teachers’ judgments (Research Question 2)

*Class teachers’* self-efficacy beliefs and self-rated emotional stability are both associated with a reduced perception of disruptions of the methodological-didactic setting, a more optimistic perception of the teacher–student relationship, and higher scores in the evaluation of their classroom management (Table 2). In contrast, there is no significant association between class teachers' self-efficacy beliefs, emotional stability, and teachers' perceptions of nonaggressive and aggressive students' disruptions. Thus, teacher-efficacy and emotional stability are associated only with domains, which from the teachers' perspective are predominantly under their control. By contrast, the perception of aggressive and nonaggressive behavior of the students, which from the teacher perspective roots in external sources, are not related to the teachers' emotional stability and self-efficacy beliefs. These results are clearly in line with Hypothesis 2a.

**Table 2:** Correlations between emotional stability, teacher efficacy, and three forms of classroom disruptions, teacher–student relationship, and classroom management from the perspectives of teachers and students

	SET	NON	AGS	REL	CLA
*Class teacher*					
EMS	− .29*	− .15	− .14	.30**	.38**
TSE	− .23*	− .12	− .01	.32**	.37**
*Subject teacher*					
EMS	− .30**	− .23*	− .37**	.16	.04
TSE	− .55**	− .48**	− .12	.43**	.08
*Students’ ratings of the class teacher*					
EMS	− .04	− .09	− .10	.04	.07
TSE	− .10	− .05	.02	.21	.15
*Students’ ratings of the subject teacher*					
EMS	− .14	− .19	− .22*	.23**	.18
TSE	− .47**	− .41**	− .30**	.38**	.25*

*Note.* EMS = emotional stability; TSE = teacher self-efficacy; SET = methodological-didactic setting disruptions; NON = nonaggressive disruptions by students; AGS = aggressive disruptions by students; REL = teacher–student relationship; CLA = classroom management; the significance calculation is based on uncorrected correlations; *p < .05; **p < .01

*Subject teachers* have only a little influence on classroom management rules of a given class and are less familiar with the individual characteristics of students. Consequently, we did not expect any relation between subject teachers' self-efficacy beliefs and their perceptions of classroom management. As expected, the results show that subject teachers' self-efficacy beliefs, as well as self-rated emotional stability, are not related to their perceptions of classroom management. For methodological-didactic setting disruptions and teacher–student relationships, we expected a similar pattern as for the class teachers. As expected, subject teachers' self-efficacy beliefs and self-rated emotional stability are both related to a reduced perception of disruptions of the methodological-didactic setting.

In contrast to the class teacher, only teacher efficacy but not emotional stability relates to a more optimistic perception of the teacher–student relationship. Contrary to the class teachers, subject teachers' emotional stability relates also to their perceptions of nonaggressive and aggressive student behavior. Teacher efficacy goes along with a more optimistic perception of nonaggressive student disruptions. Contrary to our expectations, the psychological characteristics of the subject teachers are also associated with their perceptions of students' misbehavior, going along with a reduced rating of students' disruptions. Hypothesis 2b could be confirmed only partially.

### Students’ judgments (Research Question 3)

In the next step, we examined how teachers' beliefs about their self-efficacy and emotional stability are related to students' perceptions. We hypothesized that teachers' self-efficacy and emotional stability do not merely represent self-serving bias but at least partially reflect positive classroom features.

*From the students’ perspective, class teachers’* self-efficacy beliefs and self-rated emotional stability are not related at all to their perceptions of classroom disruptions, teacher–student relationships, and classroom management (Table 3). Hypothesis 3a must, therefore, be rejected.

**Table 3:** Teacher ratings of classroom features regressed on student ratings (step 1) and additionally, teacher efficacy and emotional stability (step 2) (n = 82)

Variables	Final Model (step 2)							
	Classroom features	Psychological factors						
	*ß* SP	*ß* EMS	*ß* TSE	*F*(3/78)	*p*	*R*_2_^2^	*R*_1_^2^	*p change*
*Class teacher*								
SET	0.49***	− 0.23*	− 0.10	13.440	< .001	0.32	0.25	.012
NON	0.30**	− 0.10	− 0.07	3.455	.020	0.08	0.09	.410
AGS	0.22*	− 0.13	0.03	1.951	.128	0.03	0.04	.547
REL	0.23*	0.22	0.19	6.016	.001	0.16	0.06	.006
CLA	− 0.01	0.28*	0.27*	6.700	< .001	0.17	0.00	< .001
*Subject teacher*								
SET	0.39***	− 0.14	− 0.33**	20.571	< .001	0.42	0.31	< .001
NON	0.20	− 0.07	− 0.38**	9.411	< .001	0.24	0.12	.001
AGS	0.40***	− 0.32**	0.10	9.892	< .001	0.25	0.18	.012
REL	0.01	0.02	0.43***	6.034	.001	0.16	0.02	.001
CLA	− 0.05	0.02	0.09	0.232	.874	0.00	0.00	.723

SP = Student Perception; EMS = Emotional Stability; TSE = Teacher Self-Efficacy; *R*_2_^2^adjusted R^2^ of the second step, additionally including teachers’ self-rated emotional stability and self-efficacy; *R*_1_^2^ adjusted R^2^ of first step, including only student ratings of classroom features; p change informs if the inclusion of psychological variables (EMS and TSE) in the model explains significantly additional variance; *p < .05; **p < .01; ***p < .001. SET = methodological-didactic setting disruptions; NON = nonaggressive disruptions by students; AGS = aggressive disruptions by students; REL = teacher–student relationship; CLA = classroom management

The opposite holds for *students’ perspectives on the subject teacher.* Subject teachers’ high self-efficacy and emotional stability are associated with multiple positive effects from the students’ perspective of classroom characteristics*.* Subject teachers’ emotional stability goes along with a reduced perception of aggressive student behavior and a more optimistic evaluation of teacher–student relationships from the students’ perspective. This effect is even more apparent for self-efficacy. A strong sense of teacher self-efficacy goes along with a more positive evaluation of all dimensions of the studied classroom features from the students’ perspective.

### To what extent do psychological variables predict teacher perceptions? (Research Question 4)

In the next step, we tested to what extent psychological variables predict teacher perceptions after controlling for the students' ratings, which represent rather objective classroom features. Stepwise multiple linear regression analyses were performed to identify independent variables that contributed to teachers' perceptions of classroom features. In a first step, we entered the student ratings (representing rather objective features of the classroom) into the regression analysis. In a second step, we added teacher self-efficacy beliefs and emotional stability (which represent psychological factors). Five multiple linear regressions were calculated for each teacher category to predict three forms of classroom disruptions, teacher–student relationships, and classroom management based on student ratings and teachers' emotional stability and self-efficacy expectations.

The results in Table 3 show that *class teachers’ perceptions of classroom disruptions* can be explained predominantly by rather "objective" features of the classroom (approximated by the students' ratings). In contrast, the psychological variables of teacher self-efficacy and emotional stability contribute only a small part to the prediction of teachers' perceptions of methodological-didactic disruptions and not a specific contribution to perceptions of nonaggressive and aggressive student behavior. A different picture emerges for teacher perceptions of teacher–student relationships and classroom management. Both dimensions can be explained predominantly by psychological variables rather than “objective” classroom features. Albeit for teacher–student relationship, both predictors of emotional stability (*p* = .053) and teacher self-efficacy (*p* = .093) just miss statistical significance; together, their contribution is statistically significant (*p* = .006) to the regression. This effect is even more apparent in classroom management. Here, students' perceptions contribute only 3 per mill of the variance of the teacher rating. By contrast, self-rated emotional stability and perceived self-efficacy contribute 20.5% (*R*^2^; not adjusted) of additional variance.

*Subject teachers’ perceptions* of methodological-didactic setting disruptions and aggressive disruptions by students can be explained predominantly by rather “objective” features of the classroom. Nonaggressive student disruptions can be explained in equal parts by psychological variables and student ratings. The subject teachers’ perceptions of teacher–student relationships are almost exclusively explained by teacher self-efficacy. Surprisingly, classroom management isn’t explained by either the students’ ratings or psychological variables of the subject teacher.

Finally, we tested for *curvilinear effects* of teacher self-efficacy and emotional stability and teachers’ *gender* and *years of professional experience*. The results clearly show the linear effect of emotional stability and teacher self-efficacy. Further, the contribution of teachers’ gender and professional experience are not statistically significant to the regression equations.

## Discussion

In this study, we focused on how teachers’ self-efficacy beliefs and emotional stability are associated with teachers’ and students’ perceptions of classroom disruptions, classroom management, and teacher–student relationships.

*Class teachers’* emotional stability and a high sense of efficacy color their perceptions of their classroom management, teacher–student relationships, and methodological-didactic setting disruptions. However, class teachers' self-efficacy beliefs and self-rated emotional stability are not associated with students' perceptions of classroom disruptions, teacher–student relationships, or classroom management. Hence, students do not benefit from class teachers with high self-attributed emotional stability and a high sense of efficacy. For class teachers, optimistic beliefs about self-efficacy and emotional stability may be functional. They may operate as a filter, leading to a more optimistic perception of classroom features that are primarily under their control. Such self-serving bias may be functional for the psychological wellbeing of the teacher.

*Subject teachers’* emotional stability and teacher efficacy are associated with more favorable student perceptions of classroom disruptions, teacher–student relationships, and classroom management. In teachers with high self-rated self-efficacy and emotional stability, students perceive fewer classroom disruptions, and teacher efficacy is associated with improved teacher–student relationships. By contrast, subject teachers' self-efficacy beliefs and self-rated emotional stability are not related to their perceptions of classroom management. A possible explanation is that it is the class teacher who introduces classroom rules and takes on most of the teaching. Subsequently, the subject teacher is confronted with an already established set of norms and regulations and has only a minor influence on the topic. Besides, subject teachers spend less time with the students of a given class and are less familiar with the individual characteristics of the students. Both factors may hamper successful classroom management. Class teachers' emotional stability and self-efficacy weren't related at all to student perceptions. In contrast, subject teachers' high self-efficacy is associated with reduced classroom disruptions, good teacher–student relationships, and sound classroom management, from the students' perspective.

*To* sum up. For class teachers, a high self-rated emotional stability and self-efficacy represent something like a funhouse mirror, making them feel good about their skills. However, these positive self-evaluations are not associated with more positive student perceptions of classroom processes. Quite the contrary applies to subject teachers. Subject teachers' high self-efficacy beliefs seem to be a keystone for successful instruction, from both the teachers' and students' perspectives.

### Objective features and psychological variables

In conducting multiple regression analyses, we examined to what extent teachers’ perceptions are determined by "objective features" of the classroom or psychological variables of the teacher. The results show that psychological variables do not influence the class teachers' perception of nonaggressive and aggressive student misbehavior. On the contrary, teachers' perceptions of teacher–student relationships are determined predominantly by teachers' self-rated emotional stability and self-efficacy. The minor influence of "objective" classroom features explaining the teacher–student relationship could be partially explained by the fact that teachers rated their classes, while students rated their teachers (e.g., "I like this teacher" or "I like my class"). Concerning the teacher–student relationship, teacher and student questionnaires did not measure the same. Finally, teachers' perceptions of classroom management can be almost exclusively explained by psychological variables. Hence, for class teachers, both self-efficacy and emotional stability contribute to a more optimistic view of the teacher–student relationship and classroom management and may reflect a self-serving bias of the class teacher. Overall, in class teachers, the inclusion of self-efficacy and emotional stability explains more variance for dimensions, which are from teacher’s viewpoint predominantly under their control.

For *subject teachers*, self-efficacy seems to play a decisive role. A high sense of self-efficacy relates to a more favorable perception of teacher–student relationships, disruptions of the methodological-didactic setting, and nonaggressive disruptions by students. Emotional stability contributes to a lower teacher perception of student aggression.

This study measured *teacher self-efficacy* as a task-specific construct reflecting teachers' beliefs in coping with classroom disruptions. This rises the question, why this particular form of self-efficacy seems to be so relevant for teachers' perceptions of teacher–student relationships and classroom management. Classroom management includes all actions that teachers take to evoke a safe and stimulating learning environment. The prevention of classroom disruptions forms an essential part of classroom management. Therefore, the association between self-efficacy and classroom management is not as surprising as it might first appear.

The relation between the teacher–student relationship and teacher self-efficacy is more complex. It can be assumed that teacher self-efficacy represents a prerequisite for building successful relationships with students. Following an interpersonal approach, the teacher–student relationship can be conceptualized by two orthogonal dimensions: agency and communion [50]. Good teacher–student relationships are characterized by a high degree of communion and adequate teacher agency. Self-efficacy seems to be of particular importance for the agency dimension. A teacher can exert agency only with the belief that he or she can handle the situation.

### Limitations

This study has several limitations. First, we aimed to test the influence of psychological variables on teachers’ perceptions of classroom features. However, self-efficacy and emotional stability represent only a small subset of possible factors that may influence teachers’ perceptions. Furthermore, we conceptualized student perceptions as an *approximation* of "objective" classroom characteristics. However, in no case should student perceptions be interpreted as a portrait of "reality."

Moreover, in our study, we assessed only a single measurement point obtained from the questionnaire surveys; we are therefore limited to correlational data. Finally, the teacher efficacy scale was very short and showed low internal consistency. This was because the original study focused in the first instance on classroom disruptions.

Our study assessed teacher self-efficacy as a relatively stable disposition of the teacher as a personality trait. However, we are aware that there may be reciprocal effects between classroom features and teacher self-efficacy. In a further step, it would be interesting to investigate teacher efficacy in classroom management by adopting a longitudinal design [51], including different methodological approaches. This would permit the consideration of possible reciprocal effects and testing for reverse effects of classroom disruptions, teacher–student relationships, and classroom management on teacher self-efficacy beliefs. Students' behavior in class may function as a predictor of teachers' self-efficacy beliefs. Teacher self-efficacy and "outcomes" may affect one another reciprocally [16]. Finally, our study measured teacher efficacy at a classroom level, thereby reflecting the collective valence of teachers' sense of self-efficacy in coping with classroom disruptions of all students. However, teachers have to adopt a dual focus on the class and individual students. Research focuses typically on teachers' perceptions of the class. Zee et al. [16] argue that there may be much intra-individual variability in teachers' appraisals of individual students' behavior and self-efficacy beliefs.

## Conclusions

This study investigated how teachers' self-efficacy and emotional stability are related to teachers' and students' perceptions of the teacher–student relationship, teacher–student relationships, and classroom management. The results show a distinctive pattern for class teachers and subject teachers. In class teachers, high self-rated emotional stability and self-efficacy are associated with a more positive evaluation (compared to student ratings) of the teacher–student relationship and classroom management skills but not teacher perceptions of student misbehavior. On the contrary, subject teachers' firm self-efficacy beliefs are associated with more favorable perceptions of classroom disruptions, teacher–student relationships, and classroom management, both from the teachers' and students' perspectives.

Finally, we focus on four concrete implications for the pedagogical practice. (1) Teacher education could, instead of praising emotional stability and self-efficacy as a panacea, sensitize (prospective) teachers in a differentiated way to the dual function and limitations: (a) Both protect against subjective teacher stress (b) However, they only partially (in the case of subject teachers) contribute to an objectively measurable improvement of teaching quality. (2) School principals should ensure that the selected subject teachers have sufficiently high emotional stability and self-efficacy when filling minimal teaching positions. Because subject teachers, despite their high pedagogical competencies, face more significant challenges in classroom management than classroom teachers due to their role and reduced contact time. (3) Classroom teachers should increasingly involve subject teachers in establishing classroom rules and supporting them in difficult teaching situations. (4) Especially the classroom teachers should always make an effort to look at the lessons from the students' point of view, take the students' perspective, and critically question possible self-serving biases.

## Supplementary Information

**Additional file 1.** Classroom Disruption Questionnaire.

## Data Availability

The dataset analyzed during this study is available from the corresponding author on reasonable request.
