# Supplementary material for: Class- and subject teachers’ self-efficacy and emotional stability and students’ perceptions of the teacher–student relationship, classroom management, and classroom disruptions
Source: BMC Psychol. 2021 Jul 8;9:103. doi: 10.1186/s40359-021-00606-6 (PMC8268445; doi:10.1186/s40359-021-00606-6)
Supplement: Supplementary file 1 — Additional file 1. Classroom Disruption Questionnaire. [file 40359_2021_606_MOESM1_ESM.docx]

**English language version of the questionnaire** (Supplementary file)

|  | **Student version** | | **Teacher version** |  |
| --- | --- | --- | --- | --- |
|  | *How often does this occur in the lessons of this teacher?* | | *How often does this occur in your lessons?* |  |
| **Non-aggressive behavior of students** | | | |  |
| 1 | Some children are chatting while this teacher explains something. | | Some students are chatting during explanatory phases. |  |
| 2 | Some children chat during silent working. | | Some students chat during silent working. |  |
| 3 | Some children are occupied with completely different things (e.g. writing letters). | | Some students are occupied with completely different things (e.g. writing letters). |  |
| 4 | Some children do not listen to this teacher properly. | | Some students do not listen to me properly. |  |
| **Aggressive behavior of the students** | | | |  |
| 1 | Children beat or kick other children. | | Children beat or kick other children. |  |
| 2 | Children threaten other children. | | Children threaten other children. |  |
| 3 | Children insult or abuse other children. | | Children insult or abuse other children. |  |
| 4 | Children deliberately accuse other children even though they have done nothing. | | Children deliberately accuse other children even though they have done nothing. |  |
|  | *To what extent are these statements true?* | | *To what extent are these statements true?* |  |
| **Methodological-didactic setting disruptions** | | | |  |
| 1 | In the lessons of this teacher, there are many disturbances (e.g. interruptions and restlessness). | | In my lessons, there are many disturbances. |  |
| 2 | This teacher must often intervene to get attention. | | I have to intervene often to get attention. |  |
| 3 | For this teacher, it is easy to make the class peaceful and quiet. (inverse) | | For me, it is easy to make the class peaceful and quiet. (inverse) |  |
| 4 | When changing between different tasks, it takes a very long time with this teacher, until all children are ready again. | | When changing from different tasks, it takes a very long time until all students are ready again. |  |
| **Teacher-Student Relationship** | | | |  |
| 1 | I like this teacher. | | I like my class. |  |
| 2 | I get along well with this teacher. | | I have a good relationship with my students. |  |
| 3 | This teacher supports us when we have problems. | | I support my students when they have problems. |  |
| 4 | He/She is a good teacher. | | Overall I have a good class. |  |
| 5 | This teacher teaches in such a way that we are attentive and participate well. | | I teach so that the children are attentive are and participate well. |  |
| 6 | This teacher teaches interestingly. | | My students think I teach interestingly. |  |
| **Classroom Management** | | | |  |
| 1 | This teacher has an overview of the, what happens in class. | | I have an overview of what happens in class. |  |
| 2 | This teacher notices when children are not of the matter. | | I notice when students are not of the matter. |  |
| 3 | In case of a disturbance, this teacher will react quickly and appropriate. | | In case of a disturbance, I react quickly and appropriate. |  |
| **Teacher self-efficacy** | | | |  |
| 1 |  | | I can do a lot to prevent disruptions through adaptive classroom organization. |  |
| 2 |  | | Due to my experience with classroom disruptions, I can manage even difficult classroom situations. |  |
| 3 |  | | I am sure I can reach even disruptive students when I try. |  |
| **Emotional stability** | | | |  |
| 1 | |  | I often feel tense and nervous. |  |
| 2 | |  | I quickly resign myself to lack of success. |  |
| 3 | |  | I can handle disappointments. |  |
